# Non-Viral Nanoparticle Delivers Small Interfering RNA to Macrophages In Vitro and In Vivo

**DOI:** 10.1371/journal.pone.0118472

**Published:** 2015-03-23

**Authors:** Mei Zhang, Yunxiang Gao, Kevin Caja, Bocheng Zhao, Julian A. Kim

**Affiliations:** 1 Department of Biomedical Engineering, Case Western Reserve University, Cleveland, Ohio, United States of America; 2 Case Comprehensive Cancer Center, Case Western Reserve University, Cleveland, Ohio, United States of America; 3 Department of Macromolecular Science and Engineering, School of Engineering, Case Western Reserve University, Cleveland, Ohio, United States of America; 4 Division of Surgical Oncology, University Hospitals, Case Medical Center, Cleveland, Ohio, United States of America; University of South Florida, UNITED STATES

## Abstract

Macrophages are increasingly being viewed as therapeutic target for various cancers and many inflammatory diseases. Sequence specific gene reduction by siRNA represents an attractive approach to modulate macrophage function. However, delivery of the therapeutic siRNA into macrophages by non-viral nanoparticles has been a major technical challenge. In this study, we developed a glucan-based siRNA carrier system (BG34-10-Re-I) and demonstrated that the BG34-10-Re-I can effectively assemble siRNA into uniformly distributed nanoparticles of the novel core-shell structure. The BG34-10-Re-I/siRNA nanoparticles effectively reduced gene expression of macrophage migration inhibitory factor (MIF) in primary macrophages at both protein and mRNA level. The nanoparticles also mediated a sustained reduction of MIF within primary macrophages. Moreover, systemic injection of the nanoparticles into the Balb/c mice bearing 4T1 mammary tumors resulted in the MIF reduction in tumor-associated macrophages. Mechanistic studies demonstrated that the glucan-shell and the siRNA-core structure contribute to the effective delivery of MIF siRNA to macrophages both in vitro and in vivo. This study represents the first development of the primary macrophage MIF gene targeted non-viral nanoparticle system for both in vitro and in vivo applications.

## Introduction

Macrophages play a significant role in various inflammatory diseases and cancers and are increasingly being viewed as therapeutic target of great potential [1–9]. Sequence specific gene modification by small interfering RNA (siRNA) provides an attractive opportunity to modulate relevant gene expression in macrophages [10]. However, transfecting primary macrophage with siRNA has been a technical challenge [11]. Primary matured macrophages are end-stage cells that do not divide so that most of the siRNA delivery systems depending upon the integration into dividing cells do not work for macrophages [11]. Therefore, macrophage-targeted drug delivery system needs to facilitate an active uptake of siRNA drugs [12–14]. Furthermore, macrophages are professional phagocytes endowed with many potent degradative enzymes. These enzymes can disrupt nucleic acid integrity, making gene transfer inefficient and short-lived [11]. Therefore, macrophage-targeted drug delivery system needs to protect the integrity and stability of siRNA during delivery [12–14]. Lastly, macrophages are mediators of adaptive immune responses so that the gene of interest within macrophages needs to be manipulated for a prolonged period of time in order to effectively stimulate immune responses [8, 11]. Therefore, macrophage-targeted delivery system needs to enable a prolonged delivery of siRNA to mediate functional property [12–14].

The glucan-based materials, such as dextran and yeast glucan, have been used to deliver drugs and/or imaging agents to monocytes and/or macrophages that are accumulated in disease tissues [15–17]. This is because the glucans adopt pathogen-associated molecular pattern (PAMP) so that they can be recognized and actively internalized by macrophages via receptor-induced interaction. In previous study, we have developed a water soluble *β*-(1→3)-(1→4)-glucan (BG34) of various molecular weights (Mw) (1 kDa, 10 kDa, and 50 kDa) [18]. The BG34 of 10 kDa (BG34-10) was found to mediate the most-effective receptor-induced internalization by primary macrophages via interaction with CD11b [18]. This has led to the current development of the BG34-based nanoparticle system to deliver siRNA into the non-dividing macrophages. It has also been shown in our previous results that the macrophage receptor-induced internalization is not interfered by modifying the reducing end of the BG34-10 [18]. This provides the opportunity to conjugate functional groups to the BG34-10 and then use them to complex siRNA. In this study, we developed BG34-based carrier system and examined its ability to deliver siRNA to macrophages both in vitro and in vivo.

In this study, we used the validated sequence specific siRNA to manipulate the macrophage migration inhibitory factor (MIF) in primary macrophages. MIF has been identified as a major gene product up-regulated in disease tissue (such as tumor) where macrophages infiltrate and accumulate [19, 20]. MIF is considered a pluripotent cytokine and chemokine that plays a significant role in regulating immune responses of the tumor microenvironment [21]. Increasing evidence have suggested that MIF can function differently in intracellular and extracellular spaces of macrophages [22, 23], both of which correlate to cancer development and metastasis. Taking these into consideration, the siRNA technology demonstrates to be an advanced tool to manipulate MIF as compared to MIF antibodies or antagonists [24]. This is because the siRNA can reduce MIF at mRNA level so that both the extracellular and the intracellular MIF will be reduced. Thus, the non-viral nanoparticles targeting primary macrophage MIF gene could lead to novel therapeutic strategy for cancers and inflammatory diseases.

Intravenous injection of glucan-based materials has been demonstrated to result in the uptake of glucan, such as dextran, by circulating monocytes/macrophages with a subsequent infiltration/accumulation of these macrophages at the site of tumors or inflammatory tissues [16, 17, 25]. Such the strategy has been used to deliver drugs and/or imaging agents to macrophage-infiltrated disease tissues [26]. In this study, we evaluated our glucan (BG34-10)-based nanoparticle system for macrophage-targeted siRNA delivery in vivo using Balb/c mice bearing 4T1 mammary tumors. The 4T1 tumor has been shown to associate with a dramatic infiltration of tumor-associated macrophages (TAMs). Using the 4T1mice model, we evaluated the bio-distribution of the nanoparticles and the MIF expression in TAMs. Our results demonstrated the effective delivery of siRNA to macrophages in vivo. Therefore, this study represents the first development of the primary macrophage MIF gene-targeted non-viral nanoparticle system for both in vitro and in vivo applications.

## Materials and Methods

### Materials

The double stranded validated sequence specific MIF siRNA for murine macrophages (5’-CCGCAACUACAGUAAGCUGdTdT-3’ (sense), 5’-CAGCUUACUGUAGUUG-CGGdTdT-3’(anti-sense)), and the AF488 conjugated MIF siRNA (AF488-siRNA) for murine macrophages (AF488–5’-CCGCAACUACAGUAAGCUGdTdT-3’ (sense), 5’-CAGCUUACUGUAGUUG-CGGdTdT-3’ (anti-sense)) were custom-made by Invitrogen (Invitrogen, Calsbad, USA). The RNase-free water, RPMI-1640 medium, DMEM medium, fetal bovine serum (FBS), penicillin/streptomycin, ACK red blood cell lysis buffer, cell fixative buffer, Hoechst blue 33342, mounting agent and anti-fade reagent, HRP-conjugated anti-rabbit and anti-goat secondary antibodies, rabbit-anti-mouse AF405-conjugated F4/80 antibodies, rabbit-anti-mouse AF647-conjugated MIF antibodies, intracellular staining kit, were also purchased from Invitrogen. The Mission siRNA-Negative control SIC001 was purchased from Sigma (Sigma, St. Louis, USA) and used as a scrambled siRNA (mis-matched siRNA). The ethylenediamine (EDA), NaBH_4_, sulfur reactive mercaptoimidazole, SDS, glycerol, 2-mercaptoethanol, bromphenol blue, Tris-HCl, bafilomycin A1 and cocktail of proteinases and peptidases were purchased from Sigma (Sigma, St. Louis, USA). The amine reactive sulfo-SMCC, strong cation exchange spin column, 12% Precise protein gels were purchased from Pierce (Pierce, Rockford, USA). The Mw standard oat β-glucan of 1, 10 and 50 kDa was purchased from Putus Macromolecules (Putus, Wuhan, China). The Mw cutoff spin column (Mw cutoff = 3,000 Da) and 0.45 μm filter were purchased from Millipore (Millipore, Billerica, USA). FuGENE and Xtreme were purchased from Roche (Roche Applied Science, Indianapolis, USA). ExGene was purchased from (Fermanta, Glen Burnie, USA). The immortalized normal monkey kidney cell line Vero was purchased from American Type Tissue Collection (ATCC, Rockville, USA). The CellTiter 96 Aq_ueous_ One Solution Reagent was purchased from Promega (Promega, Madison, WI). All the ELISA kits were purchased from MER&CIEL company (www.meretciel.com). The thioglycollate was purchased from Fisher (Fisher, Leicestershire, UK). The BCA assay kit and cell lysis buffer were purchased from Thermo Scientific (Thermo Scientific, Rockford, USA).

### Preparation of the nanoparticles loaded with siRNA

The 5 μL of siRNA of three different concentrations (0.1, 1 and 2 μg/μL) was respectively added to BG34-10-Re-I solutions at N/P ratio of 1, 10 and 20. For example, for N/P = 2, the 5 μL of siRNA (0.1 μg/μL) were added to 4.6 μL of BG34-10-Re-I solution at 5.0 mg/mL, followed by vortex mixing and then 20–30 minutes incubation; for N/P = 10, the 5 μL of siRNA (0.1 μg/μL) were added to 4.6 μL of BG34-10-Re-I solution at 25 mg/mL, followed by vortex mixing and 20–30 minutes incubation; for N/P = 20, the 5 μL of siRNA (0.1 μg/μL) were added to 4.6 μL of BG34-10-Re-I solution at 50 mg/mL, followed by vortex mixing and 20–30 minutes incubation. The nanoparticle suspension was diluted by RNase free water or PBS to 500 μL to prepare nanoparticle in water or PBS, respectively.

### Transfection

The nanoparticles were directly added into the culture of macrophages. The nanoparticles containing 5 μg siRNA were used to transfect 1 × 10^6^ macrophage cells. Macrophage cultures were not washed after the nanoparticle treatments.

### Colorimetric assay

The time-dependent siRNA release from the nanoparticles under different environments was determined by colorimetric assay. At various time points, the BG34-10-Re-I/(AF488-MIF-siRNA) nanoparticles were centrifuged at 8,000 rpm for 15 minutes. One hundred μL aliquots of the supernatants were added into 96 well plates to measure fluorescence intensity of the AF488 by colorimetric assay. This was to quantify the time-dependent release of the AF488-MIF-siRNA from the BG34-10-Re-I/(AF488-MIF-siRNA) nanoparticles in water and PBS.

The time-dependent siRNA release in the presence of intracellular hydrolytic enzymes was investigated by colorimetric assay. The BG34-10-Re-I/(AF488-MIF-siRNA) nanoparticles in PBS were added by a cocktail of peptidases (Sigma) at 40 units/mL. At various time points, the nanoparticles were centrifuged at 8,000 rpm for 15 minutes. One hundred μL aliquots of the supernatant were added into 96 well culture plate to measure the fluorescence intensity of the AF488 by colorimetric assay.

The colorimetric assay was conducted in triplicates by measuring the absorbance at 520 nm using a Perkin Elmer 1420 Multilabel microplate reader. Data were analyzed using Wallac 1420 Manager Software.

### Dynamic light scattering (DLS) and Static light scattering (SLS)

The DLS and SLS measurements were performed with a Brookhaven Laser Light Scattering system with a BI200SMv2 goniometer with a vertically polarized helium-neon diode laser at a wavelength of 633 nm and a BI-9000AT digital correlator with a 125 ns initial measurement time. Samples were kept at constant temperature (25°C) for the duration of the measurements. Measurements were taken every 10° between 30° and 140°. SLS measurements were analyzed in a Berry plot to obtain the radius of gyration. The size and size distribution of the nanoparticles were determined by LLS (ALV 5000) with a vertically polarized 22-mV He-Ne laser head of 633 nm. Both the DLS and SLS measurements were conducted in triplicates.

### Transmission electron microscopy (TEM)

The morphology of the BG34-10-Re-I/(AF488-MIF-siRNA) nanoparticles were imaged using a 300 KV field-emission gun energy-filtering high resolution analytical scanning TEM Tecnai F30 ST (FEI, Hillsboro, USA). A carbon-coated 200-mesh copper specimen grid (Agar Scien-218 tific Ltd., Essex, UK) was glow-discharged for 1.5 minute. Five μL of the BG34-10-Re-I/siRNA nanoparticles were deposited on substrate, frozen by liquid nitrogen and then freeze dried for TEM imaging. No negative staining was used as that studies have shown that the negative staining could destroy the assembly of the glucan with DNA, RNA, and peptides, while samples prepared with no negative staining can generate good quality TEM images that well reflect the structure.

### Cell culture

Macrophage cell line PMJ2Rs were purchased from ATCC. Thioglycollate-elicited mouse peritoneal extrude cells were prepared according to our previous publication [18]. The adherent cells were used as primary mouse macrophage cells. All the cells were cultured in DMEM medium supplemented with 5% (v/v) FBS and penicillin/streptomycin. Cells were kept in incubator (37°C) supplemented with 5% CO_2_ and 95% humidity.

### Fluorescence microscopy

Cellular uptake of the nanoparticles loaded with the AF488-conjugated siRNA was imaged by fluorescence microscopy. The macrophages were seeded on cover slide in 6-well culture plate at 1 × 10^6^ cells/well and treated by the nanoparticles loaded with 5 μg of the AF488-conjugated siRNA. For intracellular localization of the AF488-MIF-siRNA, the macrophages were incubated with lysotracker for 30 minutes before the treatment with the BG34-10-Re-I/(AF488-MIF-siRNA) nanoparticles. After the treatment, the macrophages were washed by PBS and fixed by 1% paraformaldehyde. The fixed macrophages were incubated with Hoechst blue 33342 for 15 minutes and mounted on a glass slide using mounting and anti-fade reagent from Invitrogen. The fluorescence images were acquired on a Carl Zeiss fluorescent microscope system LSM 510 and processed by Zeiss LSM Image software (Zeiss, Jena, Germany).

### Western blot

Protein expression of MIF in the macrophages treated by PBS, BG34-10-Re-I/(AF488-scrambled siRNA) and BG34-10-Re-I/(AF488-MIF-siRNA) nanoparticles was determined by western blot in triplicates according to standard method. Rabbit-anti-mouse MIF antibody and the HRP-conjugated rabbit secondary antibody were used to detect MIF. Goat anti-mouse β-actin antibody and the HRP-conjugated goat secondary antibody were used to detect β-actin (internal control). The enhanced chemiluminescence (ECL) Plus Western Blotting Detection System (Amersham Biosciences UK Limited, Buckinghamshire, England) was used to visualize proteins according to the manufacturer’s protocols.

### Flow cytometry

The cells were treated using fixation and permeabilization solution kit according to the manufacture’s protocol (BD biosciences). The fixed cells were incubated with rabbit IgG for 30 minutes followed by an incubation with the AF405-conjugated rabbit-anti-mouse F4/80 antibody. After washed by PBS, the cells were permeabilized and then intracellular stained with rabbit IgG and then the AF647-conjugated rabbit-anti-mouse MIF antibody. All the surface and intracellular stain were conducted in dark for 30 minutes. After intracellular stain, cells were washed and re-suspended in stain buffer for FACS analysis. The FACS analysis was performed in triplicates in a BD LSR-II (Life sciences Research) flow cytometer with CellQuest software (Becton Dickinson, Mountain View, USA). Data were processed and analyzed using Winlist software (Verity House Software).

Whether the nanoparticle-mediated gene silencing within macrophages be affected by bafilomycin A1 was examined by intracellular stain of MIF followed by FACS analysis, as described above. For the study on the effect of bafilomycin A1 on nanoparticle-mediated reduction of MIF, macrophages were treated with bafilomycin A1 for 30 minutes before the nanoparticle treatments were conducted.

### Mice experiments

The 7–8 week old female Balb/c mice were maintained in accordance with the guidelines of the use and care of experimental animals and approved by Animal Research Center of Case Western Reserve University under the protocol number IACUC 2010–0127. A million 4T1 mammary tumor cells were injected to the mammary fat pad of mice. Ten days post tumor cell injection, the nanoparticles loaded with the AF488-MIF-siRNA were injected into the mice every other day for 7 days via tail vein. The dose of MIF siRNA was 0.8 mg/kg/day. PBS solution and the nanoparticles loaded with the scrambled siRNA were used as controls. Each group includes 8 mice and repeated twice for statistics.

In order to determine the biodistribution of the AF488-MIF-siRNA after the i.v. injection of nanoparticles, we euthanized mice at 2, 4 and 12 hours after the injection and harvested tumor, kidneys, livers, spleen, lungs, heart, brain and blood. The tumor, organ and blood samples were homogenized to collect supernatants. Fluorescence intensity of the AF-488 of the supernatants was quantified by colorimetric assay in triplicates.

In order to determine the silencing efficacy in vivo, 3 days after the i.v. injection of nanoparticles we euthanized the mice and harvested the tumors. The tumors were digested by digestive enzyme cocktail (Sigma) to obtain single cell suspension. The obtained cells were stained by AF405-conjugated F4/80 antibody followed by intracellular stain with the AF647-conjugated MIF antibody. FACS analyses were conducted in triplicates according to the method described above.

## Results

### Development and characterization of the BG34-10-based carrier system (BG34-10-Re-I) and its complexation with siRNA

The amines and imidazoles were conjugated to the reducing end of the β-glucan (BG34-10) (Fig. A—(A) in S1 Dataset). The obtained product is labeled as BG34-10-Re-I. The amines and imidazole were introduced to the BG34-10 as positively charged molecules to enable the complexation with negatively charged siRNA molecules. Molecular weight (Mw) and Mw distribution of the BG34-10 and the BG34-10-Re-I were characterized by high performance size exclusion chromatographic (SEC), demonstrating that they are homogeneous glucan materials with uniformly distributed Mw (Fig. A—(B) in S1 Dataset). Chemical structures of the BG34 and the BG34-10-Re-I were characterized by FTIR and ^1^H NMR, shown in Fig. A—(C) and (D) in S1 Dataset, respectively, demonstrating the successful conjugation of amines and imidazole.

Further, the BG34-10-Re-I was complexed with siRNAs at three different concentrations and three different N/P ratios. The sequence validated siRNA targeting macrophage migration inhibitory factor (MIF-siRNA) was used. The MIF-siRNA was added into the aqueous solution of the BG34-10-Re-I using micrometer-sized syringe pump under vortex mixing, followed by LS analysis at 90° to quickly measure the size of any possibly formed particles (Table 1).

**Table 1:** SLS measurement of the BG34-10-Re-I/(AF488-MIF-siRNA) complex in PBS.

siRNA concentration	BG34 derivatives	N/P	Size Measurement by LLS
0 μg / μL	BG34-10-Re-I		18.2 ± 4.7 nm
0.1 μg / μL	BG34-10-Re-I	2	15.1 ± 3.7 nm
		10	14.9 ± 5.1 nm
		20	17.1 ± 2.7 nm
0.5 μg / μL	BG34-10-Re-I	2	16.3 ± 2.5 nm
		10	139.1 ± 13.5 nm
		20	512.7 ± 46.7 nm
5 μg / μL	BG34-10-Re-I	2	11.3 ± 9.9 nm
		10	21.3 ± 7.5 nm
		20	33.8 ± 7.1 nm

Results indicated that the nanoparticles of ∼139.1± 13.5 nm and 512.7 nm were observed when the MIF siRNA of 0.5 μg/mL were added to the BG34-10-Re-I solution at N/P = 10 and = 20, respectively, suggesting the complexation between the MIF siRNA and the BG34-10-Re-I. The obtained nanoparticle complexes were analyzed by DLS and SLS and results were summarized in Table 2.

**Table 2:** Structural characteristics of the BG34-10-Re-I/(AF488-MIF-siRNA) complex.

Complex	N/P	R_G_	R_H_	PDI	R_G_/R_H_	Structure
BG34-10-Re-I/siRNA	10	42.4	61.9	0.24	0.685	Core-shell
BG34-10-Re-I/siRNA	20	65.5	52.6	0.31	1.25	Large particle

**Fig 1:**
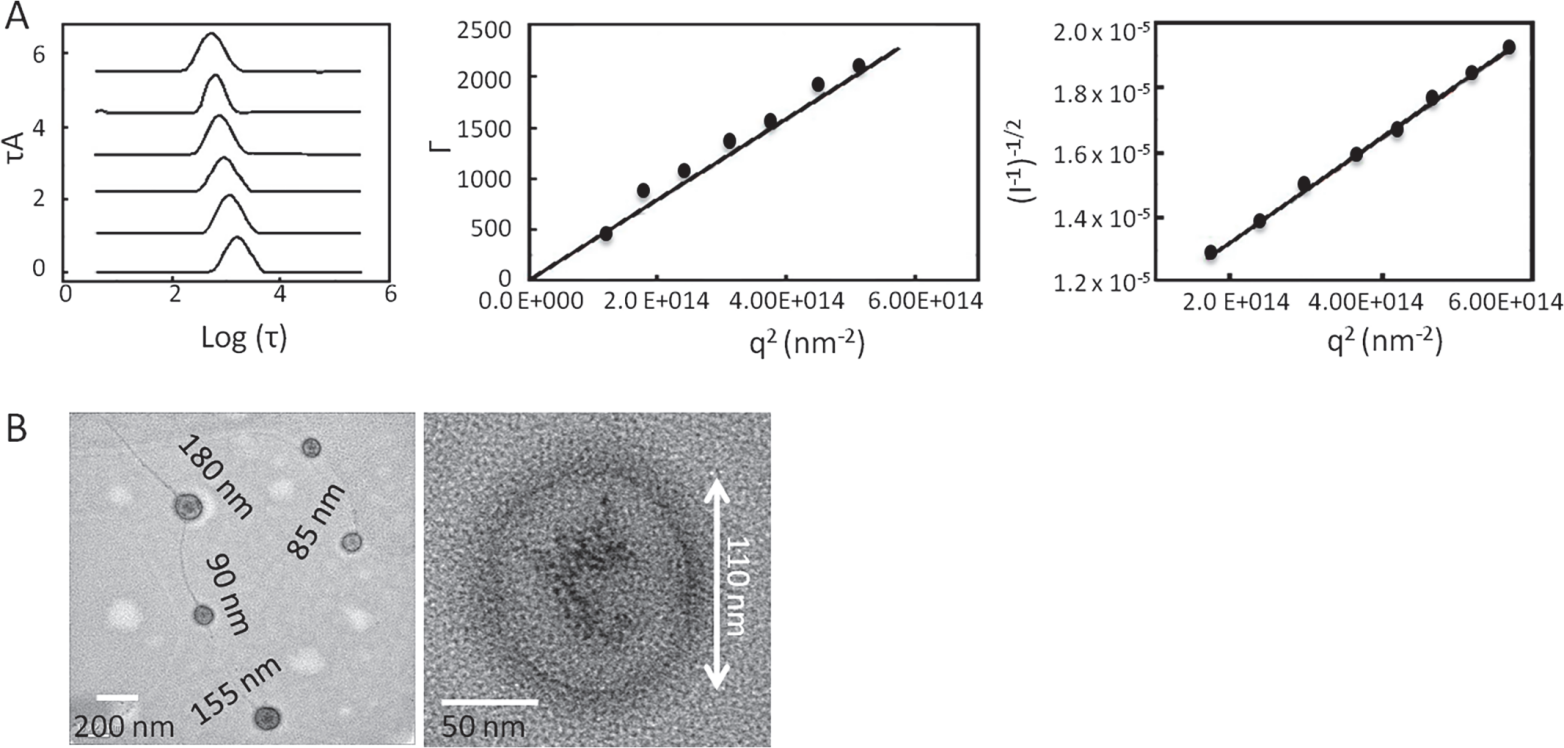
Size and morphology characterization of the BG34-10-Re-I/(AF488-MIF-siRNA) nanoparticle at N / P = 10. **A**. DLS and SLS characterization of the nanoparticles; **B**. TEM characterization of the nanoparticles.

The DLS and SLS data were shown in Fig. 1 A. The particles showed narrow size-distributions over multiple scattering angles. The values of the decay constant (Γ) for all the particles exhibited linear *q* ^2^ dependence, which is the hallmark of diffusive motion related to the presence of spherical particles. Additionally, Berry plots generated from the SLS data over a broad angle range displayed a linear relationship between (I^-1^)^-1/2^ and *q* ^2^. Based on the R_G_/R_H_ ratio, the nanoparticle complexed at N/P = 10 exhibited characteristics of core-shell structure, whereas the particles complexed at N/P = 20 exhibited relatively large particle structure.

In order to determine size and morphology of the nanoparticles, TEM imaging was performed. The TEM images indicated that the nanoparticle exhibits a dark core and a light shell (Fig. 1 B). It has been shown in literature that the water-soluble cationic glucan materials highly swell in water with conjugated water molecules bound to the glucan chain [27–29]. After freeze-drying the nanoparticles, the conjugated water molecules that swelled on the glucan were lyophilized; therefore, the electron beams could easily pass through, which resulted in a light region in the TEM images [28, 29]. By contrast, the siRNA is composed of phosphate groups of high density, which can prevent the electron beam from easily passing through so that exhibit a dark region in TEM images [28, 29]. These previous observations well explain the siRNA-core and the glucan-shell structure of the BG34-10-Re-I/(AF488-MIF-siRNA) nanoparticle system.

Noted that there is a filament structure in the TEM image that seemed to connect the nanoparticles. Indeed, the filament structures have been widely observed for polysaccharide samples. The filament structures of 3–5 nm (or 5–8 nm) are considered to be the bundle structure of polysaccharide chains that have been repeatedly observed on the freeze-dried polysaccharide samples by SEM or TEM [30].

### The BG34-10-Re-I/(AF488-MIF-siRNA) nanoparticles mediated effective internalization by primary macrophages

**Fig 2:**
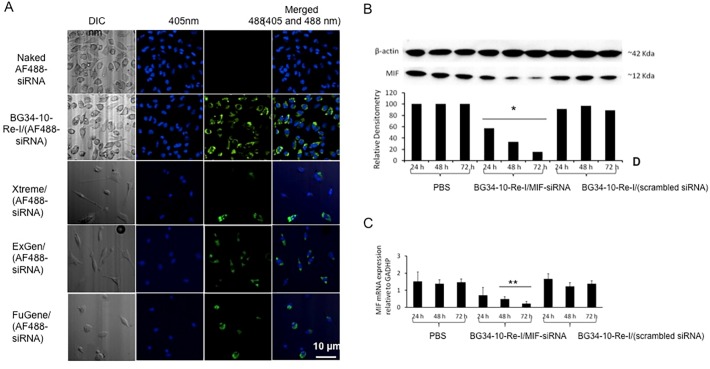
Transfection of primary macrophages using the BG34-10-Re-I/(AF488-MIF-siRNA) nanoparticles. **A**. Confocal fluorescence microscopic images of the macrophages after the addition of the BG34-10-Re-I/(AF488-MIF-siRNA) nanoparticles. The DIC image showed the three-dimensional surface geometry of macrophage cells. The naked FITC-siRNA served as negative control. The FITC-siRNA complexed by commercial carriers Xtreme, Exgene and Fugene served as positive control. Macrophages were stained by Hoechst blue and imaged at 405 nm. The FITC-siRNA within macrophages was imaged at 488nm. Images representative of three independent experiments with similar results are shown. **B.** Western blot measurement of the MIF protein in the macrophages treated by the BG34-10-Re-I/(MIF siRNA) nanoparticles at 24, 48 and 72 hours. MIF protein expression was normalized to the expression of β-actin. Macrophages treated by PBS and the BG34-10-Re-I/(scrambled siRNA) nanoparticles served as negative controls. **C**. qRT-PCR analysis of the MIF mRNA in the macrophages treated by the BG34-10-Re-I/(MIF siRNA) nanoparticles at 24, 48 and 72 hours. MIF mRNA expression was normalized to the expression of housekeeping gene GAPDH. Macrophages treated by PBS and BG34-10-Re-I/(scrambled siRNA) nanoparticles served as negative controls. Both the western blot and the qRT-PCR was conducted in triplicates.

The BG34-10-Re-I/(AF488-MIF-siRNA) nanoparticles were co-cultured with Balb/c mice bone marrow-derived and LPS-activated macrophages to examine the macrophage uptake of the AF488-MIF-siRNA in vitro, using the naked AF488-siRNA and the commercially available cationic gene carrier systems as controls (Fig. 2 A). After co-culture, the macrophages were stained by Hoechst blue and imaged by confocal fluorescence microscopy. Results revealed that the macrophages did not spontaneously uptake the naked AF488-conjugated siRNA. The macrophages could uptake the AF488-MIF-siRNA complexed by three commercially available carriers (Fugene, Xtreme and ExGene). However, these three commercial carriers associated with reduction of the total number of the macrophages due to their cellular toxicity (Fig. B in S1 Dataset). In contrast, the BG34-10-Re-I/(AF488-MIF-siRNA) nanoparticles mediated effective internalization by the macrophages and showed no cellular toxicity (Fig. B in S1 Dataset).

### The BG34-10-Re-I/(AF488-MIF-siRNA) nanoparticles mediated effective MIF reduction in primary macrophages

Having determined the dose of siRNA that mediated the effective MIF reduction in the macrophages (Fig. C in S1 Dataset), we used the nanoparticles loaded with 5 μg AF488-MIF-siRNA to transfect 1 × 10^6^ macrophages. After the transfection, the macrophages were determined by western blot to quantify the MIF protein expression (Fig. 2 B) and by qRT-PCR to quantify the MIF mRNA expression (Fig. 2 C). The macrophages treated with PBS and the nanoparticles loaded with the scrambled siRNA served as controls. Results of the western blots demonstrated that the macrophages treated by PBS and the nanoparticles loaded with the scrambled siRNAs express MIF protein (∼ 12 Kda) at 24, 48, and 72 hours, indicating that the MIF protein expression in macrophages is not affected by these controls. In contrast, the macrophages treated by the BG34-10-Re-I/(AF488-MIF siRNA) nanoparticles demonstrated a reduction of the MIF protein by 43%, 67% and 81% at 24, 48 and 72 hours, respectively, revealing the effective reduction of MIF protein. Results of the qRT-PCR demonstrated that, as compared to macrophages treated by controls, the macrophages treated by the BG34-10-Re-I/(AF488-MIF siRNA) nanoparticles resulted in the effective reduction of the MIF mRNA by over 50% at 24, 48 and 72 hours. To date, non-viral nanoparticle system has not been reported to silence a wild gene (not genetically transfected gene with high expression background such as GFP or luciferase) in primary macrophages by over 50%. Our BG34-10-Re-I/(AF488-MIF siRNA) nanoparticle system could effectively deliver siRNA into primary macrophages and silence both protein and mRNA expression of the wild target gene by over 65% at 72 hours (Fig. 2 B and C).

### The BG34-10-Re-I/(AF488-MIF-siRNA) nanoparticles effectively delivered the AF488-conjugated siRNA into cytoplasm

**Fig 3:**
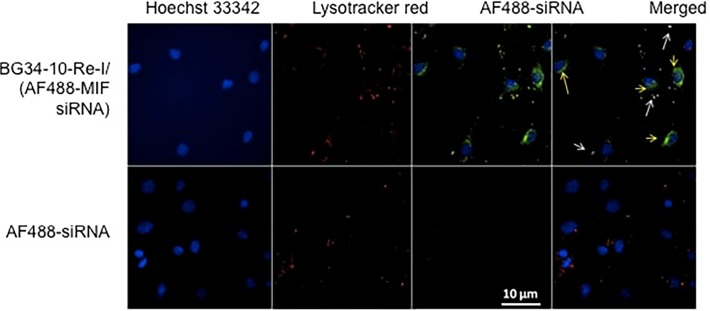
Confocal fluorescence microscopic images of the PMJ2R macrophage cells after treatment with the BG34-10-Re-I/(AF488-MIF-siRNA) nanoparticles and lysotracker red. The lysotracker was used to label endosome-lysosome compartments. Naked AF488-siRNA served as control. Images representative of 3 independent experiments with similar results are shown.

In order to determine whether the BG34-10-Re-I/(AF488-MIF-siRNA) nanoparticles can deliver the siRNA into cytoplasm where the RNAi machinery works, we examined the co-location of the AF488-MIF-siRNA with sub-cellular compartments within macrophage cells (Fig. 3). In this study, lysotracker was used to label the lysosome compartments of cells. Since the lysotracker has shown limited ability to label the non-dividing cells, we used macrophage cell line PMJ2R to examine the co-localization. Results demonstrated that the macrophage cells did not spontaneously uptake the naked AF488-siRNA. In contrast, the macrophages treated by the BG34-10-Re-I/(AF488-MIF-siRNA) nanoparticle demonstrated the presence of the AF488-MIF-siRNAs in both the AF647-labeled lysosome compartments (arrow indicated) and cytoplasm (Fig. 3). This suggested that the nanoparticles effectively deliver the AF488-MIF-siRNA into the cytoplasm within macrophage cells.

### The BG34-10-Re-I/(AF488-MIF-siRNA) nanoparticles mediated persistent reduction of the target gene MIF within primary macrophages

**Fig 4:**
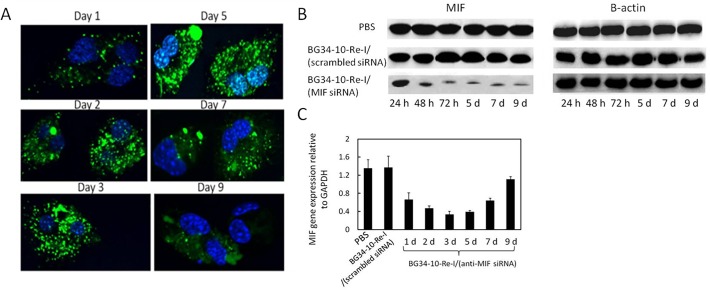
The AF488-siRNA delivery and the MIF expression within primary macrophages after the addition of a single dose of the nanoparticles. **A**. Confocal fluorescence microscopic images of the macrophages culture after the addition of a single dose of nanoparticles. Cultures were not washed after nanoparticle treatment. Data representative of 3 independent experiments with similar results are shown. **B**. Western blot analysis of MIF protein expression in the macrophages. The MIF protein expression was normalized to the expression of *β*-actin. **C**. qRT-PCR analysis of MIF mRNA expression in the macrophages. The MIF mRNA was normalized to the expression of GAPDH. For **B** and **C**, PBS and the BG34-10-Re-I/(scrambled siRNA) nanoparticles served as negative controls. Both the western blot and the qRT-PCR were conducted in triplicates.

Since our nanoparticle system showed no cytotoxicity to macrophages, the macrophages cultures were not washed after nanoparticle treatment. This is different from other practice that needs to wash away toxic nanoparticles from cultures. In this study, after the addition of a single dose of the nanoparticles loaded with the AF488-MIF-siRNA, the primary macrophage were tested to examine MIF expression. At various time points, macrophage internalization of the nanoparticles loaded with the AF488-MIF-siRNA was imaged by confocal fluorescence microscopy, suggesting increased internalization of siRNA over time (Fig. 4 A). The green fluorescence was seen in macrophages for up to 7 days and faded on day 9. The MIF protein and mRNA expression in macrophages at various time points were examined by western blotting (Fig. 4 B) and qRT-PCR (Fig. 4 C). As compared to the macrophages treated by the controls, the macrophages treated by the BG34-10-Re-I/(MIF siRNA) nanoparticles demonstrated the reduction of the MIF protein for up to 9 days (Fig. 4 C) and mRNA for up to 7 days (Fig. 4 D).

### Intravenous injection of the BG34-10-Re-I/(AF488-MIF-siRNA) nanoparticles into the 4T1 tumor-bearing Balb/c mice resulted in accumulation of the AF488-MIF-siRNA in tumor

**Fig 5:**
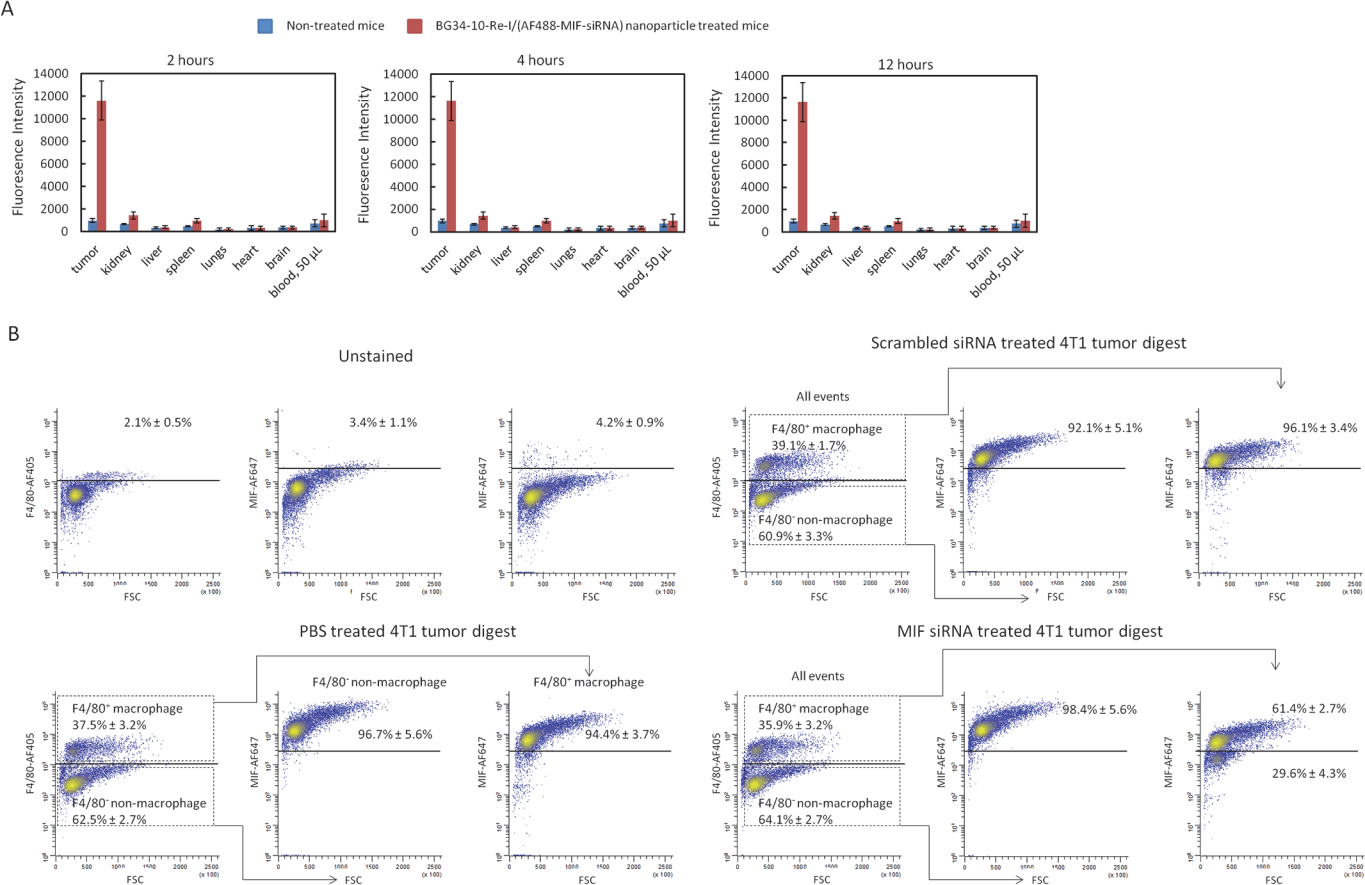
In vivo evaluation of the BG34-10-Re-I/(AF488-MIF-siRNA) nanoparticles. The nanoparticles loaded with the AF488-MIF siRNA were i.v. injected to Balb/c mice bearing day-10 4T1 tumor (∼ 5 x 7 mm). The i.v. injection was administered every other day for 7 days. **A**. Biodistribution of the AF488-MIF-siRNA at 2, 4 and 12 hours after the i.v. injection of the BG34-10-Re-I/(AF488-MIF-siRNA) nanoparticles. **B**. FACS analysis on the MIF protein expression in the F4/80 positive tumor-associated macrophages after the i.v. injection of the BG34-10-Re-I/(AF488-MIF-siRNA) nanoparticles. Mice treated with the nanoparticles loaded with the scrambled siRNA served as controls. Each group includes 8 mice and repeated twice.

In order to examine the in vivo bio-distribution of the nanoparticles, we tested the fluorescence intensity of the AF488-MIF-siRNA in various Balb/c mice tissues at three different time points (2, 4 and 12 hours). Concentration of the AF488-MIF-siRNA in tumor, blood, heart, liver, spleen, lung, kidney and brain tissue were measured by quantifying the fluorescence intensity using colorimetric assay. The PBS treatment was used as control to quantify auto-fluorescence. High-level accumulation of the AF488-MIF-siRNA was detected in 4T1 tumors, indicating that the i.v. injection of the nanoparticles result in a specific accumulation of the AF488-MIF-siRNA within tumors. Accumulation of the AF488-MIF-siRNA in other organs was much lower than that in tumors, indicating a low systemic dissemination (Fig. 5 A).

### Intravenous injection of the BG34-10-Re-I/(AF488-MIF-siRNA) nanoparticles into the tumor-bearing Balb/c mice resulted in MIF reduction in tumor-associated macrophages within 4T1 tumor

The BG34-10-Re-I/(AF488-MIF-siRNA) nanoparticles were injected into Balb/c mice bearing established 4T1 tumor via tail veins every other day for 7 days. PBS and the BG34-10-Re-I/(scrambled siRNA) nanoparticles served as controls. Three days after the injection, the mice were euthanized to harvest 4T1 tumors from the control and the treatment groups. The 4T1 tumor cell digestions were prepared and surface-stained by the AF405-conjugated F4/80 (AF647-F4/80) antibodies followed by permeabilization and intracellular stain with the AF647-conjugated MIF antibodies (Fig. 5 B). Results of the FACS analysis demonstrated that, as compared to the controls, ∼47% of the F4/80^+^ TAM showed reduced MIF protein expression after the treatment with the BG34-10-Re-I/(MIF-siRNA) nanoparticles. This demonstrated that the i.v. injection of the BG34-10-Re-I/(MIF-siRNA) nanoparticles result in the effective MIF protein reduction in TAMs within 4T1 tumor microenvironment.

## Discussion

Glucan-based biomaterials such as dextran and chitosan have been used to develop gene drug carrier systems due to the excellent biocompatibility and biodegradability [31]. While others focus on the development of the glucan-based gene carrier systems for complexation or condensation of large DNA plasmids to silence target genes, we developed the BG34-10 glucan-based cationic gene carrier system for the complexation of siRNA. The DNA plasmids consist of over 1000 base pairs, which require the delivery system to provide 4–5 amine functions per molecules to enable the complexation [32, 33]. However, the high payload of amines on gene carrier systems has been discovered to cause cytotoxicity to cells both in vitro and in vivo [34]. In contrast, the siRNA only consist of 19–21 base pairs, which greatly reduces the strength needed for cationic gene carrier system interaction and complexation of siRNA [35]. In this study, we conjugated amines and imidazoles to the reducing end of the BG34 glucan of relatively low average molecular mass (10 kDa, ∼20–30 glucose molecules) (Fig. A in S1 Dataset). Although the low number of amine per glucan molecule does not enable effective complexation of large DNA plasmid (data not shown), it effectively enables the complexation of the AF488-conjugated MIF-siRNA (Table 1, 2 and Fig. 1). More importantly, the low number of amines per glucan molecule caused no cytotoxocity to the primary macrophages, which has been observed in other commercialized gene carrier systems (Fig. B in S1 Dataset).

Our results show that the BG34-10-Re-I/(AF488-MIF-siRNA) nanoparticles can mediate internalization by the primary macrophages (Fig. 2A), deliver the siRNA to the cytoplasm regions (Fig. 3), reduce the target gene MIF at both mRNA and protein level (Fig. 2 B), and prolong the MIF reduction for up to 9 days (Fig. 4). These results revealed the high efficiency of the nanoparticles for delivering the siRNA molecules to non-dividing primary macrophages for the effective manipulation of a wild type gene MIF. Note that this is different from the manipulation of a genetically engineered gene such as GFP or luciferase that shows an extremely high background of gene expression.

The capability of the BG34-10-Re-I/(AF488-MIF-siRNA) nanoparticles of mediating the specific and active internalization by primary macrophages (Fig. 2 A) is believed to attributed to the BG34-10 glucan shell (Fig. 1). The BG34-10 has been found to mediate an active receptor-induced internalization by primary macrophages [18].

The capability of the BG34-10-Re-I/(AF488-MIF-siRNA) nanoparticles to effectively deliver the AF488-MIF-siRNA into cytoplasm (Fig. 3) is very important because RNAi machinery works in the cytoplasm [36]. Generally, after the nanoparticles are internalized by cells, intracellular trafficking begins in the endosomes, which are acidified (pH 5.4–6.0) by membrane H^+^ ATPases [36]. The endosomes further transfer the content into lysosomes, which are further acidified (pH ∼5.4) and contain various nucleases that can degrade siRNAs [36]. Our observation that the siRNA can be delivered to cytoplasmic regions within macrophages implies that the BG34-10-Re-I/(AF488-MIF-siRNA) nanoparticle can facilitate escape of the siRNA from the endosome-lysosome compartments.

**Fig 6:**
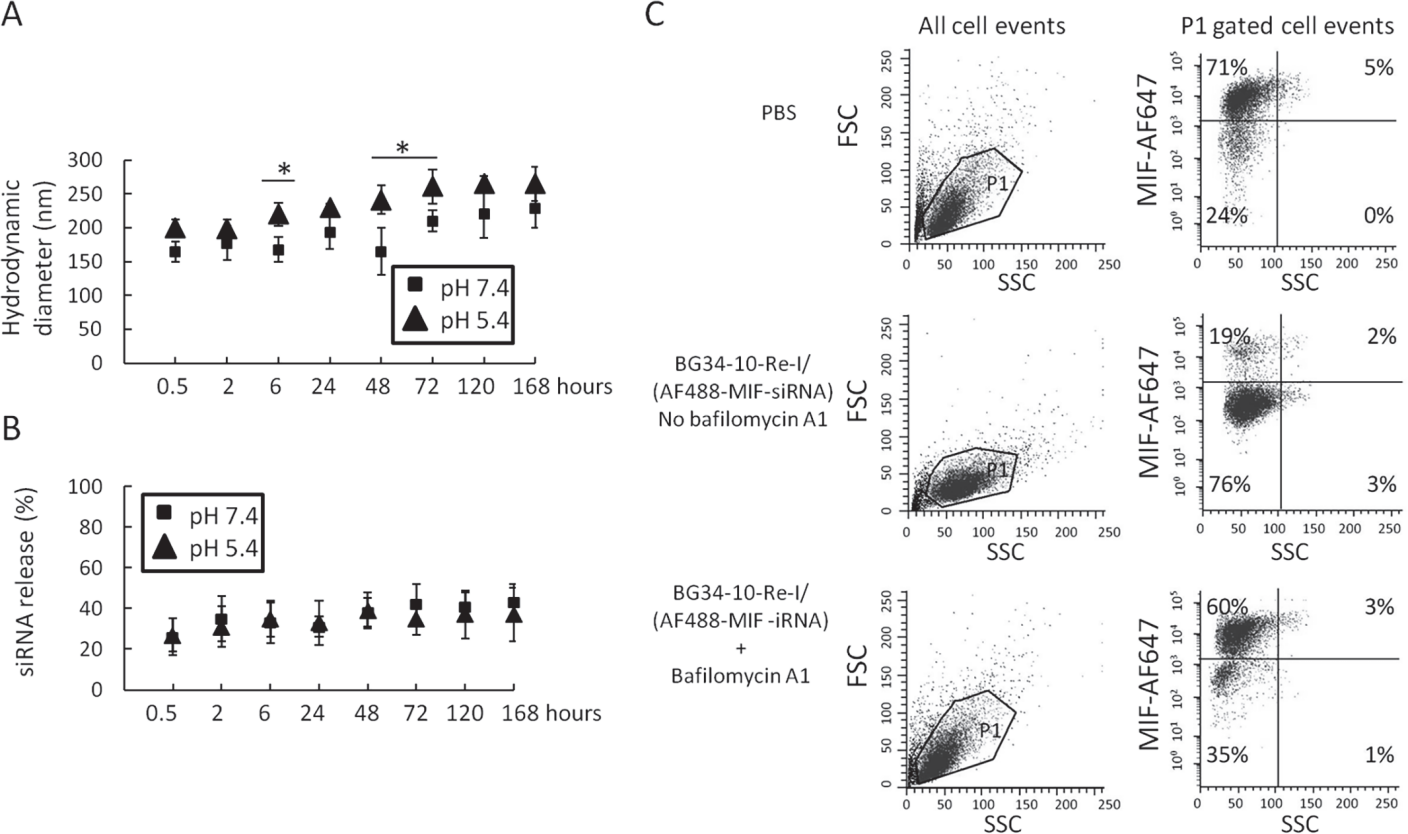
The BG34-10-Re-I/(AF488-MIF-siRNA) nanoparticle-mediated siRNA delivery in macrophages. **A**. LLS measurement of the size of the BG34-10-Re-I/(AF488-MIF-siRNA) nanoparticles at pH 7.4 and 5.4. **B**. Colorimetric assay of the AF488-siRNA release from the BG34-10-Re-I/(AF488-MIF-siRNA) nanoparticles at pH 7.4 and 5.4. **C**. FACS analysis on the intracellular MIF protein expression in PMJ2R macrophages treated by the BG34-10-Re-I/(MIF siRNA) nanoparticles in the presence and absence of bafilomycin A1. The macrophages treated by PBS served as negative control. The macrophages treated by the BG34-10-Re-I/(AF488-MIF-siRNA) nanoparticles in the absence of bafilomycin A1 showed effective reduction of MIF and served as positive control. The macrophages treated by the BG34-10-Re-I/(AF488-MIF-siRNA) nanoparticles in the presence of bafilomycin A1 showed that the BG34-10-Re-I/(AF488-MIF-siRNA) nanoparticle-mediated reduction of MIF protein in primary macrophages was attenuated. The LLS, colorimetric and FACS analysis were all conducted in duplicates.

In order to examine whether the BG34-10-Re-I/(AF488-MIF-siRNA) nanoparticles can facilitate the escape of the siRNA from lysosomes to cytoplasm, we tested stability of the nanoparticles by examining the nanoparticle size (Fig. 6 A) and the siRNA release at different pHs (Fig. 6 B). The results demonstrated stability of the nanoparticles at endosomal-lysosomal pH, suggesting that the nanoparticles are capable of protecting the siRNA from degradation at acidic lysosomal pH. Further, we tested the effect of bafilomycin A1 on the nanoparticle-mediated MIF reduction within macrophages by FACS (Fig. 6 C). The BG34-10-Re-I carrier system contains imidazole molecule (Fig. A-(A) in S1 Dataset), which has been discovered to absorb protons under acidic pH 5.4–6.5 [37, 38]. The Bafilomycin A1 is an antibiotic that can act as a specific inhibitor of vacuolar-type H^+^-ATPase in cells to prevent the re-acidification of subcellular compartments such as endosomes and lysosomes [39]. Here, we used bafilomycin A1 to test whether the BG34-10-Re-I/(AF488-MIF-siRNA) nanoparticles delivered siRNA to cytoplasm via mediating re-acidification in the endosome-lysosome compartments to disrupt these compartments. If the nanoparticles do, the addition of the bafilomycin A1 that inhibits re-acidification will attenuate the MIF gene reduction caused by the nanoparticles. Our results demonstrated that the bafilomycin A1 attenuated the nanoparticle-mediated MIF reduction in macrophages (Fig. 6 C), suggesting the capability of the nanoparticles to mediate re-acidification in the endosomes-lysosome compartments.

**Fig 7:**
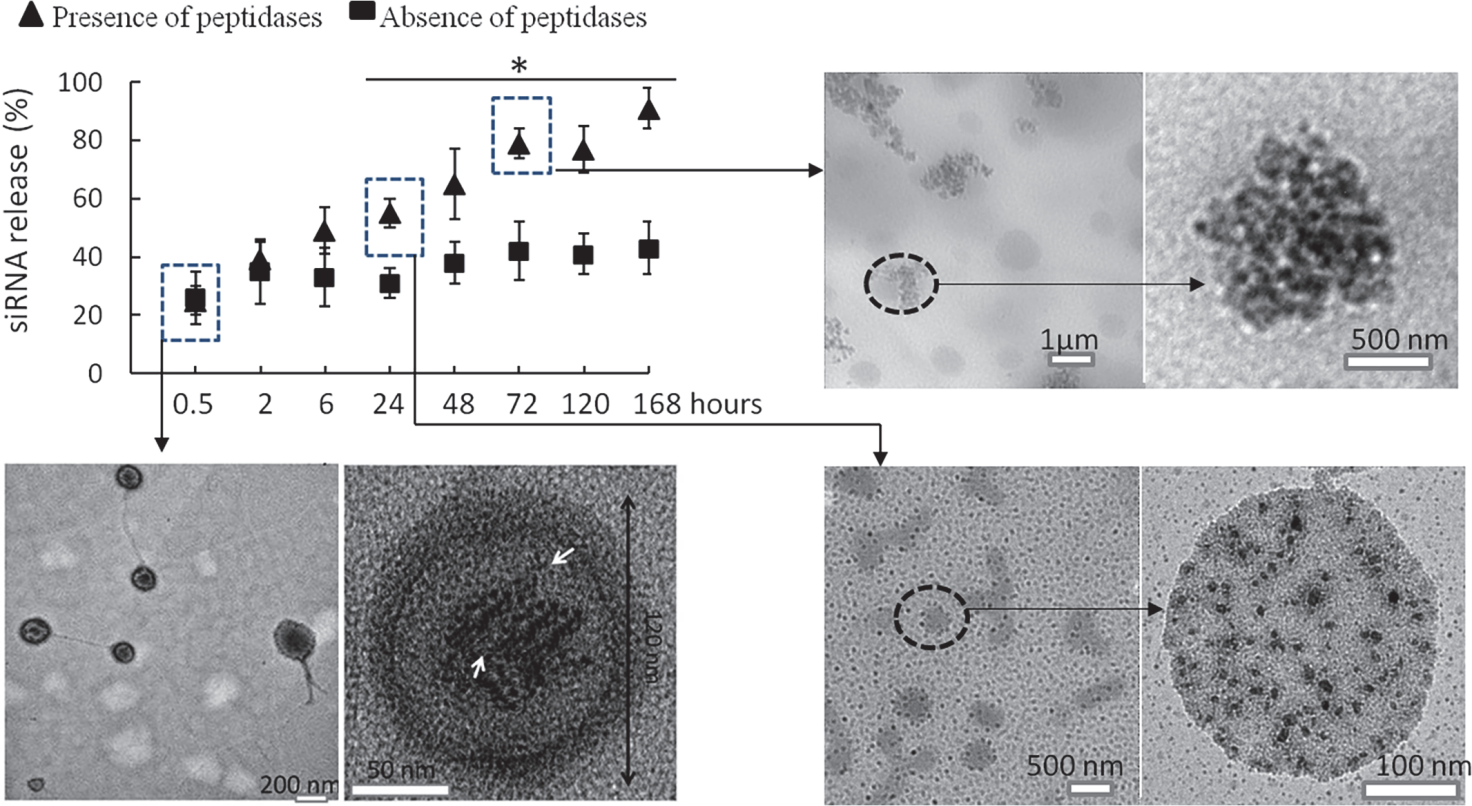
Colorimetric assay on the siRNA release from the BG34-10-Re-I/(AF488-MIF—siRNA) nanoparticles in the presence and absence of peptidases. At different time points, TEM was performed in triplicates to examine the size and morphology of the nanoparticles. *, P < 0.05.

One of the challenges of manipulating target genes with siRNA is the transient effect [36, 38]. In this study, after the addition of a single dose of the nanoparticle, the primary macrophages demonstrated reduced expression of the MIF mRNA and protein for up to 9 days (Fig. 4). Recent studies have provided insights into the kinetics of the siRNA-mediated gene silencing, which indicates that the duration of gene silencing from 1 week to 3 weeks are considered persistent silencing [38]. Although mechanism of the persistent MIF gene silencing needs further investigation, we believed that the siRNA-core and glucan-shell structure of the nanoparticles make significant contribution to the persistent reduction of MIF. The core-shell nanoparticles demonstrated high stability and low toxicity so that they do not need to wash away from cultures. The increased internalization of siRNA by macrophages over time in Fig. 4 A may reflect the continuous internalization of nanoparticles from medium. In cytoplasm, the BG34-10-Re-I/(AF488-MIF-siRNA) nanoparticles may undergo time-dependent degradation because the glucan shell is composed of the biodegradable BG34-10 glucan and the glucan-amine conjugates. Glucans have been shown to slowly degrade within cells upon oxidation [40], while glucan-amine conjugates have been found to slowly digest by cytoplasmic peptidases [41]. Our examination of the kinetics of the nanoparticles in the presence of a peptidase cocktail revealed a time-dependent release of the AF488-MIF-siRNA (Fig. 7). This is associated with the dissociation of the nanoparticle core-shell structure (Fig. 7, TEM photographs). These results demonstrated that the core-shell structure contributes to the time-dependent siRNA release and the subsequent prolonged reduction of MIF mRNA and protein within primary macrophages.

More importantly, our results showed that the BG34-10-Re-I/(AF488-MIF-siRNA) nanoparticles could systemically deliver the siRNA to TAMs and reduce the MIF protein level in vivo (Fig. 5). Similarly, the dextran coated nanoparticle systems have been discovered to be capable of mediating active and specific uptake by macrophages in vivo due to their pathogen-associated molecular pattern (PAMP) [42]. The intravenous injection of the dextran-coated iron oxide nanoparticles can result in the uptake of these nanoparticles by circulating monocytes/macrophages [25, 26]. The circulating monocytes/macrophages then infiltrate to disease tissues such as tumors so that the iron oxide nanoparticles can be specifically delivered to macrophages at the disease tissues [23]. This explains why the BG34-10-Re-I/(AF488-MIF-siRNA) nanoparticles composed of the PAMP glucan (B34–10) shell can lead to the effective delivery of the siRNA to macrophage-infiltrated 4T1 tumors in mice with minimal accumulation in organs such as liver, kidney and spleen. Kupffer cells in liver, monocytes/macrophages in lymph nodes and spleen are part of reticulo-endothelial system to perform phagocytosis of large particles of over 200 nm [40]. Nanoparticles of ∼100 nm have been demonstrated to effectively escape in vivo non-specific uptake by reticulo-endothelial system [43]. Our nanoparticles exhibit 80–120 nm diameters, which may explain the low accumulation in livers and spleen in mice.

The *in vivo* and *in vitro* deliveries of genetic materials to primary macrophages are significantly important for the development of novel therapeutic strategies. In this study, our results demonstrated for the first time that the i.v. injection of the currently developed non-viral nanoparticles can effectively reduce the MIF expression in primary macrophages both in vitro and in vivo. Since MIF has been identified as a gene up-regulated in tumor-promoting macrophages and associated with tumor progression and metastasis [44–46], the development of the non-viral nanoparticle system may lead to innovative therapeutic strategies targeting tumor microenvironment. Furthermore, in vitro silencing of a specific gene in primary macrophages will have significant impact for many other investigational and clinical applications. For example, the nanoparticle can be used to silence specific disease-related genes in primary macrophages ex vivo and adoptively transferred to develop macrophage-based adoptive immunotherapy. Lastly, the nanoparticles may also be used to silence other genes of interest in macrophages in order to study the functions of these genes in cancers and inflammatory diseases.

## Conclusions

This study develops a glucan-based carrier system (BG34-10-Re-I) that can complex siRNA into uniformly distributed nanoparticles with novel core-shell structure. The BG34-10-Re-I/siRNA nanoparticles can effectively deliver siRNA into primary macrophages and reduce MIF protein and mRNA with high efficiency both in vitro and in vivo. This nanoparticle system can be applied for macrophage-targeted siRNA therapy for many inflammatory diseases and cancers that develop and progress in association with a dramatic macrophage infiltration.

## Supporting Information

S1 Dataset (DOCX)
