# Supplementary material for: Non-Viral Nanoparticle Delivers Small Interfering RNA to Macrophages In Vitro and In Vivo
Source: PLoS One. 2015 Mar 23;10(3):e0118472. doi: 10.1371/journal.pone.0118472 (PMC4370462; doi:10.1371/journal.pone.0118472)
Supplement: S1 Dataset — (DOCX) [file pone.0118472.s001.docx]

**SUPPPORTING INFORMATION**

U*Synthesis and purification of the BG34-10-Re-I.*

Conjugation of the amines and imidazole to the reducing end of the BG34-10 is shown in Figure A - (A). Aqueous solution of the BG34-10 (1 mg/mL, 100 mL) was reacted with ethylenediamine (EDA, 100 mL) overnight at room temperature (r.t.). Equal volume of ethanol was added to precipitate the BG34-10-Re-amine. The BG34-10-Re-amine was sequentially washed with 70% and 100% ethanol and then reduced by NaBH_4_. The BG34-10-Re-amine was suspended in dry DMSO (1 mg/mL) and reacted with sulfo-SMCC (8 mg/mL) at r.t. for 8 hours. After ethanol precipitation, the BG34-10-Re-SMCC was dried and then re-suspended in DMSO (1 mg/mL) to react with sulfur reactive mercaptoimidazole (10 mg/mL) at r.t. overnight. After ethanol precipitation, the precipitate was purified using Pierce strong cation exchange spin column according to the producer’s instruction. The material retained on the membrane absorber of column was eluted. The elution was neutralized and centrifuged in Mw cutoff spin column (Mw cutoff = 3,000 Da) to remove salts and small molecule substances. The desalted and concentrated solutions were lyophilized and labeled as BG34-10-Re-I.

*Structural characterization of the BG34-10-Re-I.*

Mw and Mw distribution (MwD) of the water soluble BG34-10 and the BG34-10-Re-I were characterized by high performance size exclusion chromatography (SEC) in connection with laser light scattering (LS) detector at 90^o^C and 632 nm and refractive index detector (RI) (TriSEC, Triple Detector system, Model Dual 250, Viscotek, Houston, USA) (Figure A – (B)). One hundred μl sample solution at 100 µg/mL was loaded onto the SEC column and led to the LLS detector for instantaneous measurement of the scattering intensity and the RI detector for the measurement of the concentration. The HPSEC system (Shimadzu Scientific Instruments, Columbia, USA) was equipped with OHpak KB-806M (Shodex, Tokyo, Japan) and Ultrahydrogel linear (Waters, Milford, USA) columns, and calibrated by Mw standard OBG-500 of 50 Kda. The eluent was 0.1M NaNO_3_ containing 0.03% (w/w) NaN_3_ at a flow rate of 0.7 ml/min. The TriSEC software of Viscotek was used for data acquisition and to calculate Mw and MwD.

FTIR spectra of the BG34-10 and the BG34-10-Re-I were obtained using a Perkin-Elmer System 2000 (Perkin-Elmer, Norwalk, Connecticut) (Figure A - (C)). The dried samples were ground with KBr powder and pressed into pellets for FTIR measurements. ^1^H NMR spectra of the BG34-10 and the BG34-10-Re-I were obtained using Bruker Avance 400 spectrometer equipped with a TXI-xyz three-gradient probe. Exchangeable protons removed by repeatedly suspending samples in D_2_O and lyophilizing (Figure A – (D)). ^1^H was measured using the Bruker standard pulse sequence with a frequency of 5000 Hz in 64 K complex data points. Relaxation delay was 5T1 to ensure accurate signal integration. Prior to the Fourier transformation, four-times zero filling and TRAF function were used to reduce the noise.

*Cytotoxicity of the BG34-10-Re-I/siRNA nanoparticles.*

Cytotoxicity of the nanoparticles loaded with AF488-MIF-siRNA on macrophages was quantified by MTS assay in triplicates (Figure B). 1 × 10^5^ cells in 96-well plate were treated by BG34-10-Re-I/(AF488-MIF-siRNA), Xtreme/(AF488-MIF-siRNA), Fugene/(AF488-MIF-siRNA) and ExGene/(AF488-MIF-siRNA) nanoparticles, respectively, in triplicates. After 24, 48, or 72 hours, CellTiter 96® Aqueous One Solution Reagent was added to each well according to the manufacturer’s instructions. After 4 hours in culture at 37^o^C, the cells were quantified for the absorbance (Abs) at 490nm using a Perkin Elmer 1420 Multilabel microplate reader. The data were analyzed using Wallac 1420 Manager Software (Perkin Elmer, Waltham, USA). The cytotoxicity of various treatments is calculated as following:

Cytotoxicity = [(Abs(PBS)-Abs(treatment))/ Abs(PBS)] × 100

Results of supplementary Figure 2 revealed that the BG34-10-Re-I/(AF488-MIF-siRNA) nanoparticles show a much lower cytotoxicity than the complexes formed between the siRNA and the various commercialized gene carrier system including Fugene, Xtreme and Exgene.

*Dose-dependent gene silencing by the BG34-10-Re-I/(AF488-MIF-siRNA) nanoparticles*.

Dose-dependent gene silencing mediated by the BG34-10-Re-I/siRNA nanoparticles was characterized by intracellular stain of the target gene followed by FACS analysis (Figure C). After various treatments, the macrophages were permeabilized using Fix/Perm kit according to the manufacture’s protocol (BD Biosciences), and then intracellular-stained using rabbit-anti-mouse AF647 conjugated MIF antibody. Rabbit IgG was used as isotype control. After staining, the fixed macrophages were quantified by FACS to determine the MIF protein expression. The FACS analysis was performed in BD LSR-II (Life sciences Research) flow cytometer in triplicates and data were acquired using CellQuest software (Becton Dickinson, Mountain View, USA). The data were processed and analyzed using Winlist software (Verity House Software). The results suggested that the 5 µg siRNA can effectively silence MIF gene expression in a million primary macrophages.

**Supplementary Figure Legends**

Figure A. The development and characterization of the non-viral siRNA carrier system BG34-10-re-I. (A). The synthetic route for BG34-1-Re-I, BG34-10-Re-I and BG34-50-Re-I. The cationic amine and imidazole were conjugated to the reducing end of glucan via crosslinker SMCC. (B). The SEC profiles of BG34-10 and BG34-10-Re-I. (C). The FTIR spectra of BG34 and BG34-10-Re-I. (D). The 1H NMR spectra of BG34-10 and BG34-10-Re-I.

Figure B. The anti-proliferative effect of BG34-10-Re-I/siRNA, Xtreme/siRNA, Fugene/siRNA and Exgene/siRNA complexes on macrophages. PBS served as negative control. High anti-proliferative effect indicated a high cytotoxicity to macrophages.

Figure C. Dose-dependent reduction of MIF protein in primary macrophages by FACS. Unstained macrophages served as negative control. PBS-treated and alexo red conjugated MIF antibody-stained macrophages served as positive control. Data representative of 2 independent experiments with similar results are shown. PBS-treated macrophages expressed MIF protein by 63.1%. The 0.5 µg and 5 µg siRNA led to the MIF protein reduction in a million macrophages from 63.1% to 40.3% and to 9.1%, respectively.


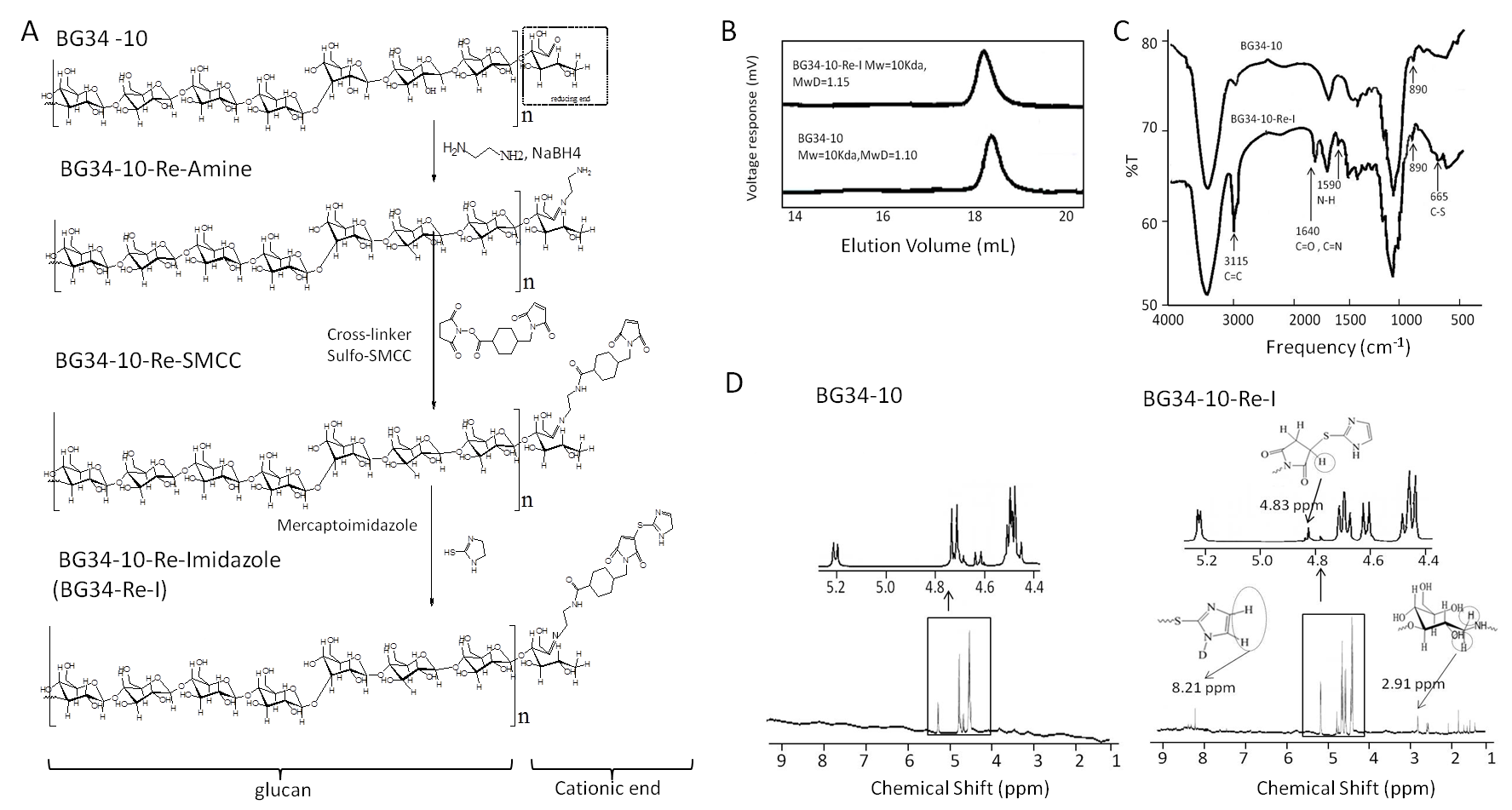


**Figure A**. The development and characterization of the non-viral siRNA carrier system BG34-10-re-I. (**A)**. The synthetic route for BG34-1-Re-I, BG34-10-Re-I and BG34-50-Re-I. The cationic amine and imidazole were conjugated to the reducing end of glucan via crosslinker SMCC. (**B)**. The SEC profiles of BG34-10 and BG34-10-Re-I. (**C)**. The FTIR spectra of BG34 and BG34-10-Re-I. (**D)**. The 1H NMR spectra of BG34-10 and BG34-10-Re-I.

**
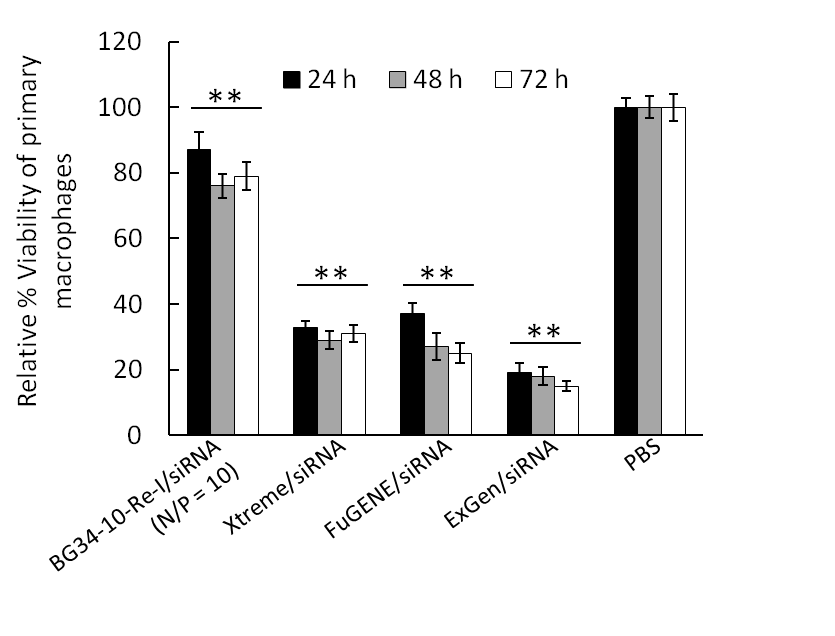
Figure B.** The anti-proliferative effect of BG34-10-Re-I/siRNA, Xtreme/siRNA, Fugene/siRNA and Exgene/siRNA complexes on macrophages. PBS served as negative control. High anti-proliferative effect indicated a high cytotoxicity to macrophages.


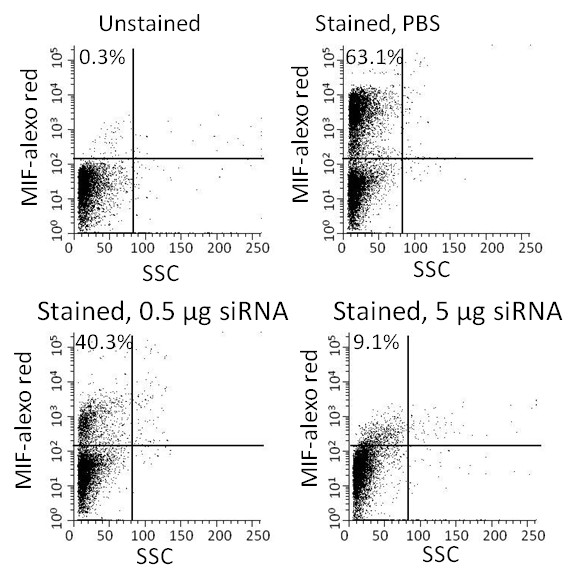


**Figure C.**  Dose-dependent reduction of MIF protein in primary macrophages by FACS. Unstained macrophages served as negative control. PBS-treated and alexo red conjugated MIF antibody-stained macrophages served as positive control. Data representative of 2 independent experiments with similar results are shown. PBS-treated macrophages expressed MIF protein by 63.1%. The 0.5 µg and 5 µg siRNA led to the MIF protein reduction in a million macrophages from 63.1% to 40.3% and to 9.1%, respectively.
